# Prosthetic treatment patterns in the very old: an insurance database analysis from Northeast Germany

**DOI:** 10.1007/s00784-020-03264-x

**Published:** 2020-04-17

**Authors:** Fabian M. Hempel, Joachim Krois, Sebastian Paris, Florian Beuer, Adelheid Kuhlmey, Falk Schwendicke

**Affiliations:** 1 Department of Operative and Preventive Dentistry, Charité – Universitätsmedizin Berlin, corporate member of Freie Universität Berlin, Humboldt-Universität zu Berlin, and Berlin Institute of Health, Berlin, Germany; 2 Department of Prosthodontics, Geriatric Dentistry and Craniomandibular Disorders, Charité – Universitätsmedizin Berlin, corporate member of Freie Universität Berlin, Humboldt-Universität zu Berlin, and Berlin Institute of Health, Berlin, Germany; 3 Institute of Medical Sociology and Rehabilitation Science, Charité – Universitätsmedizin Berlin, corporate member of Freie Universität Berlin, Humboldt-Universität zu Berlin, and Berlin Institute of Health, Berlin, Germany

**Keywords:** Access, Geriatrics, Gerodontology, Health services research, Prosthetics, Prosthodontics

## Abstract

**Objectives:**

We assessed dental prosthetic services utilization in very old Germans.

**Methods:**

A comprehensive sample of 404,610 very old (≥ 75 years), insured at one large statutory insurer (Allgemeine Ortskrankenkasse Nordost, acting in the federal states Berlin, Brandenburg, Mecklenburg-Vorpommern), were followed over 6 years (2012–2017). Our outcome was the utilization of prosthetic services, in total and seven subgroups: (1) Crowns/partial crowns, (2) fixed dental prostheses (FDPs), (3) partial removable prostheses (RDPs), (4) full RDPs, (5) temporary services, (6) relining/rebasing/repairing/extending RDPs, (7) repairing FDPs. Association of utilization with (1) gender, (2) age, (3) region, (4) social hardship status, (5) ICD-10 diagnoses and (6) German diagnoses related groups (G-DRG) was explored.

**Results:**

The mean (SD) age of the sample was 81.9 (5.4) years; mean follow-up was 1689 (705) days. The mean utilization of any prosthetic service was 27.0%; the most often utilized service type were total RDPs (13.2% utilization), crowns (8.1%), and partial RDPs (7.1%). Utilization decreased with age for nearly all services (except relining/rebasing/repairing/extending RDPs). Utilization of prosthetic services was significantly higher in Berlin and most cities compared with rural municipalities and in individuals with common, less severe conditions according to ICD-10 and DRGs compared with life-threatening conditions or dementia. In multivariable analysis, gender (OR; 95% CI: 0.95; 0.93–0.98), social hardship status (1.19; 1.17–1.21), federal state (Brandenburg 0.57; 0.56–0.59; Mecklenburg-Vorpommern: 0.66; 0.64–0.67) and age significantly affected utilization (0.95; 0.95–0.95/year).

**Conclusions:**

Patient-related and healthcare factors determine the utilization of prosthetic services in very old Germans. Interventions to maintain sufficient prosthetic care up to high age are required.

**Clinical significance:**

The utilization of prosthetic services in the very old in Northeast Germany showed significant disparities within populations and service types. There seems to be great need to better understand the drivers of utilization, and to develop and evaluate interventions to maintain sufficient prosthetic care up to high age.

**Electronic supplementary material:**

The online version of this article (10.1007/s00784-020-03264-x) contains supplementary material, which is available to authorized users.

## Introduction

Over the past 20 years, dental health in most high-income countries has significantly improved in children, adolescents, and adults, with a reduced number decayed or filled teeth; this came with a concomitantly reduced number of missing teeth in adults and the elderly [1, 2]. In the latter group, edentulism has become a rather infrequent phenomenon, as demonstrated by data from the United States [3, 4], Finland [5], Australia [6], Sweden [7] or Germany [2]. The observed differences in morbidity impact on the resulting treatment needs.

These treatment needs have been measured mainly for children and adults, with data on the very old, defined as those aged 75 years or older, being scarce. This group, however, is an increasingly relevant one, as it is growing in absolute numbers to due population aging, and as the higher number of retained teeth in the elderly come with possibly higher needs for maintenance (via oral hygiene or dental therapy). Especially in the very old, this maintenance does not necessarily seem to be given [8–10]. Especially those requiring assistance (the frail, the disabled, and the systemically sick) suffer from poor oral health [11].

Understanding the reasons for this poor oral health is required if health services are to be aligned for improving the oral health of the very old and, specifically, those requiring assistance. Claims data may be used to build some of this understanding. While such data are prone to a range of limitations, like selection bias (those insured at a specific insurance will not be fully representative for the whole population, except when dealing with generalized mandatory insurances), confounding bias, misclassification bias, and claims data not necessarily reflecting needs-adequate therapy, they offer a range of advantages: (1) Claims data yield information on groups which are otherwise not easily included in epidemiological studies. Especially the very old and, within this group, the very sick are under-represented in most studies on oral health available. (2) Claims data are usually also “big data”, yielding robust sample sizes and statistical power, and come at limited efforts and costs. (3) The data represent everyday care; they do not suffer from recollection or otherwise reporting bias and have a high generalizability in their respective (healthcare) setting [12, 13].

In the present study, we aimed to use claims data from large German public health insurance in Northeast Germany to assess prosthetic services utilization in the very old. We hypothesized that utilization differed according to age, general health, socioeconomic status, and geographically.

## Methods

### Study design

This cohort study builds on routinely collected claims data from a statutory (public) health insurance in Germany. Individuals aged 75 years or older from one large insurer, the AOK Nordost, were followed over 6 years (2012 to 2017). The AOK Nordost is a regional branch of the Allgemeine Ortskrankenkasse (AOK), acting mainly in the Northeast of Germany in the federal states of Berlin, Brandenburg and Mecklenburg-Vorpommern. The study reporting follows the RECORD statement [14].

### Setting

The AOK Nordost insures around 1.8 million individuals from the described three federal states. Insured individuals may, however, also move into other areas of Germany, which is why analyses with a geographical focus included only individuals living in these federal states throughout the follow-up period. The area of interest includes the German capital, Berlin, and two rural states, Brandenburg and Mecklenburg-Vorpommern, with only few cities larger (> 70,000 inhabitants). All three are considered economically weak in comparison with most other parts of Germany.

Data for this study were claims data, including claims from 1.1.2012 to 31.12.2017, i.e., stretching over 6 years. Data were routinely collected and provided under ethical approval in a pseudonymized form using a data protection cleared platform via the scientific institute of the AOK Nordost, the GEWiNO.

### Participants and sample size

A comprehensive sample of very old, aged 75 years or above, insured with the AOK Nordost in 2012, was drawn and followed over 6 years. No further eligibility criteria were defined. Follow-up data was provided by the AOK Nordost (see below). Variable ascertainment was only possible via insurance base data and claims data. The database had been curated for plausibility at GEWiNO and once more by the study team. No formal sample size estimation was performed given this being a comprehensive sample.

### Variables

Our outcome was the relative utilization (in % of the population) of prosthetic services. Utilization was measured by year, and absolute numbers of individuals using prosthetic services were also assessed.

Within the statutory German insurance, prosthetic services are provided based on prosthetic treatment plans (Heil- und Kostenplan), which are submitted for approval prior to service provision. Planned services fall into seven categories, which define the monetary value the statutory insurance will contribute to the expected prosthetic treatment costs, called Festzuschüsse (fixed insurance subsidies) [15]. Fixed insurance subsidies are provided for (1) Crowns/partial crowns, which are defined as prosthetic therapy in Germany, (2) fixed dental prostheses (FDPs), (3) partial removable dental prostheses (RDPs), (4) full RDPs, (5) temporary prosthetic services, (6) relining, rebasing, repairing or extending RDPs, (7) repairing FDPs. There are subgroups of fixed insurance subsidies depending on the number of missing teeth and the specific indicated treatment efforts. After approval of the treatment plan, dentists perform the planned treatments, and then claim specific provided treatments using the fee items catalogs of the German insurance [15, 16]. Patients are then paying the difference between the agreed fixed insurance subsidy (paid by the insurance) and the overall sum of claimed prosthetic treatment costs out of their own pocket. For a minority of patients with very low income (< 1246 Euro/month per capita in 2019; www.aok.de), hardship protection is provided, with the respective fixed insurance subsidy being doubled, thereby avoiding any out-of-pocket-expenses in most cases. Within the present study, fixed insurance subsidies were used to define prosthetic services. Analyzes were performed for total/any utilization (a patient utilizing any of the described seven fixed insurance subsidies) and stratified according to specific services (fixed insurance subsidies).

As this is the first detailed analysis on prosthetic treatment patterns in the very old in Northeast Germany, we provided largely descriptive analyzes. The utilization of prosthetic services was assessed according to following independent variables: (1) gender (male/female), (2) age (in years) in each year of follow-up, and (3) region; we used municipalities (Landkreise) as regional units, mainly as on a lower (more granular) spatial level only few individuals were retained in some areas. Municipalities included the capital Berlin (with over 3.5 million inhabitants), medium-sized cities (70,000–200,000 inhabitants), and rural areas, some of them only thinly inhabited. (4) Social hardship status (as described above), (5) ICD-10 diagnoses, derived from outpatient diagnostic data, (6) inpatient hospital diagnoses and treatments, derived from German diagnoses related groups (G-DRG). The G-DRGs classify diseases in groups of similar medical pathogenesis, characteristics, and treatment complexity, and are mainly used for reimbursement reasons (hospitals provide claims based on G-DRG). Only the 25 most frequently recorded ICD-10 and DRG codes were used.

### Data sources and access

The data used for this study were provided by the GEWiNO using a data protection approved data storage and analysis platform (SAHRA - Smart Analysis Health Research Access) after required cleaning and consistency controls. The data was pseudonymized, and included individuals’ age, gender, social hardship status, spatial code (Gemeindeschlüssel, allowing re-classification into municipalities) of their place of living, utilized fixed insurance subsidies for each year 2012–2017, as well as ICD-10 codes and DRGs for each year, among further variables. Comparability of data between different years and data consistency was given.

### Bias

A comprehensive sample had been used, and neither participants nor providers were aware that the collected claims data will be used for routine data analyses later on. Given that data were used pseudonymized for scientific purposes only, this was acceptable. The data collection is not prone to selection and detection bias. However, given this being claims data from only one insurance, the overall population of very old Germans differ, and data may be affected by biases associated with claims data, as laid out above and in the discussion. No further measures against these biases could or were taken.

### Statistical analyses

The statistical analysis was performed on a sample (*n* = 404,610) of the database provided by AOK Nordost. The only inclusion criterion was that an individual had to be insured in the year 2012 and had to be aged 75 years or above at this point. For the descriptive analysis of utilization of prosthetic services, we considered an individual to have consumed a particular service if at least once during the period 2012 to 2017 the provision of such a service was claimed. Descriptive statistics of age groups were computed based on the age distribution in the year 2012. The social hardship status was assessed in the same manner as the utilization of prosthetic services; an individual was assigned to having a social hardship if the individual was assigned to this status at least once during the period 2012 to 2017. For geographical analysis we excluded all individuals that relocated from one of the federal states (Berlin, Brandenburg, and Mecklenburg-Vorpommern) to another federal state, thereby decreasing the sample size to 390,044. However, we did not correct for relocations within the three federal states listed above.

For each particular outpatient diagnosis (ICD-10 codes) and inpatient hospital diagnosis and treatment (G-DRGs), we first summed up all claims and ranked them from most to least frequent. We then selected the 25 most frequent diagnoses each (in total 50) and computed for each of them the number of individuals that were assigned to having a diagnosis, respectively, prosthetic treatment, during the period 2012 to 2017. The individuals were thereafter stratified with respect to the utilization of prosthetic services.

We applied logistic regression, a method to model a binary outcome variable as a linear combination of predictor variables. The response variable was the utilization of any type of prosthetic services claimed by an individual at least once in the year 2012. As predictor variables we included age, gender, being deceased, social hardship status, federal state (note that we allowed the category ‘other’ for relocated individuals) and further 47 outpatient and inpatient hospital diagnosis variables (note that we excluded the variables E77I, I68D, and F62 owing to non-convergence of the likelihood maximization algorithm), all of them being referring to the year 2012. All analyzes, modeling and visualization were performed using Python (version 3.7, available at http://www.python.org) and auxiliary modules from its scientific computing ecosystem.

## Results

The number of insured individuals within AOK Nordost was 1,756,086 in 2018. In our study, 404,610 very old (75 years or older) individuals were sampled and followed up for 6 years. 173,733 of these did not survive follow-up; the mean follow-up was 1689 days (standard deviation SD: 705). The population was imbalanced with regard to gender (females were nearly twice as frequent as males) and age (with the majority being 75–84 years old). About one third lived in Berlin, the other two thirds in the more rural Brandenburg and Mecklenburg-Vorpommern. Social hardship status was claimed by 194,318 (48%) individuals at least once during the follow-up period (Table 1).

**Table 1:** Sample characteristics (N; %) from Northeast Germany. Total, male and female population aged 75 years or older, in 5-years age bands and according to federal state

Covariate	Group	Total	Any utilization	Crowns	FDPs	Partial RDPs	Total RDPs	Temporary	RDP repair etc.	FDP repair etc.
All		404,610 (100.0)	109,252 (27.0)	32,612 (8.06)	7934 (1.96)	28,535 (7.05)	53,210 (13.15)	15,578 (3.85)	29,363 (7.26)	1002 (0.25)
Gender	male	134,909 (33.3)	37,936 (34.7)	12,572 (38.6)	3005 (37.9)	10,297 (36.1)	18,137 (34.1)	5512 (35.4)	9144 (31.1)	330 (32.9)
	female	269,702 (66.7)	71,316 (65.3)	20,040 (61.4)	4929 (62.1)	18,238 (63.9)	35,073 (65.9)	10,066 (64.6)	20,219 (68.9)	672 (67.1)
Age group	75–79	162,367 (40.1)	56,555 (51.8)	20,409 (62.6)	5286 (66.6)	16,633 (58.3)	26,537 (49.9)	8529 (54.8)	13,932 (47.4)	533 (53.2)
	80–84	126,146 (31.2)	33,208 (30.4)	8898 (27.3)	2006 (25.3)	8302 (29.1)	16,887 (31.7)	4619 (29.7)	8764 (29.8)	303 (30.2)
	85–89	73,964 (18.3)	14,237 (13.0)	2759 (8.5)	546 (6.9)	2880 (10.1)	7192 (13.5)	1868 (12.0)	4539 (15.5)	131 (13.1)
	90–94	33,233 (8.2)	4480 (4.1)	515 (1.6)	89 (1.1)	656 (2.3)	2252 (4.2)	492 (3.2)	1754 (6.0)	32 (3.2)
	95–99	7152 (1.8)	679 (0.6)	28 (0.1)	7 (0.1)	62 (0.2)	303 (0.6)	64 (0.4)	322 (1.1)	2 (0.2)
	100–104	1675 (0.4)	91 (0.1)	3 (0.0)	0 (0.0)	2 (0.0)	38 (0.1)	6 (0.0)	51 (0.2)	1 (0.1)
	105–109	73 (0.0)	2 (0.0)	0 (0.0)	0 (0.0)	0 (0.0)	1 (0.0)	0 (0.0)	1 (0.0)	0 (0.0)
Social hardship status	no	210,292 (52.0)	52,610 (48.2)	18,129 (55.6)	4799 (60.5)	14,930 (52.3)	25,668 (48.2)	8322 (53.4)	9642 (32.8)	605 (60.4)
	yes	194,318 (48.0)	56,642 (51.8)	14,483 (44.4)	3135 (39.5)	13,605 (47.7)	27,542 (51.8)	7256 (46.6)	19,721 (67.2)	397 (39.6)
Federal state	Berlin	122,454 (30.3)	41,468 (38.0)	14,812 (45.4)	4375 (55.1)	11,547 (40.5)	19,261 (36.2)	6380 (41.0)	11,928 (40.6)	532 (53.1)
	Brandenburg	159,925 (39.5)	38,253 (35.0)	10,870 (33.3)	2225 (28.0)	10,367 (36.3)	19,108 (35.9)	5136 (33.0)	8434 (28.7)	309 (30.8)
	Mecklenburg	107,665 (26.6)	25,278 (23.1)	5932 (18.2)	1097 (13.8)	5677 (19.9)	12,882 (24.2)	3366 (21.6)	7454 (25.4)	124 (12.4)
	Others	14,566 (3.6)	4253 (3.9)	998 (3.1)	237 (3.0)	944 (3.3)	1959 (3.7)	696 (4.5)	1547 (5.3)	37 (3.7)

**Fig. 1:**
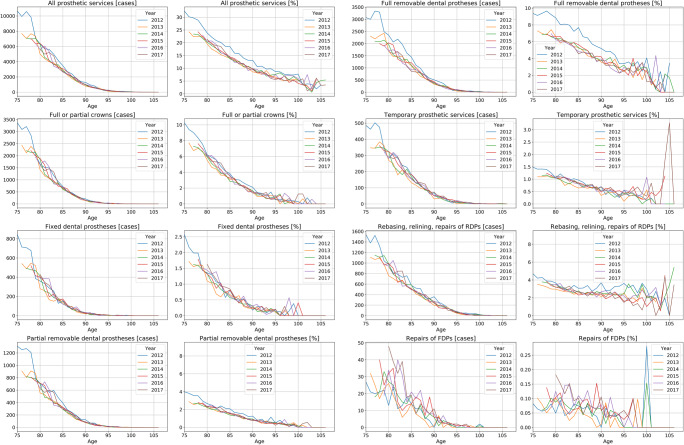
Absolute (cases) and relative (in %) utilization of prosthetic services by the very old in Northeast Germany. Total (any) utilization and specific services utilization is shown. Individuals available in 2012 of all ages from 75 years upwards (blue line) were followed over 6 years until 2017 (black line), i.e., the 75-years in 2012 are the 76-years in 2013, etc. (which is why the lines start further to the right with longer follow-up)

The mean (SD) total utilization (utilization of any prosthetic service) was 27.0%. Utilization was balanced between genders. It decreased over-proportionally with age (Fig. 1), e.g., a 75-years-old showed a utilization of 32%, indicated by the blue line in Fig. 1, which decreased to 21% at age 80 years, indicated by the brown line.

The most often utilized service type was total RDPs (13.2% utilization), crowns (8.1%), and partial RDPs (7.1%) and the repairs of RDPs (7.3%). Decreases with age (Fig. 1) were services-specific. Utilization of crowns was 10% in those aged 75 years in 2012, but only 2% for those aged 90 years. A similar decrease was observed for partial RDPs. The decrease was more pronounced for FDPs (from 2.5% to 0.3%), and less pronounced for full RDPs (from 9% to 4.5%). Relining/rebasing/repairs of RDPs and repairs of FDPs did not show a significant decrease with age.

**Fig. 2:**
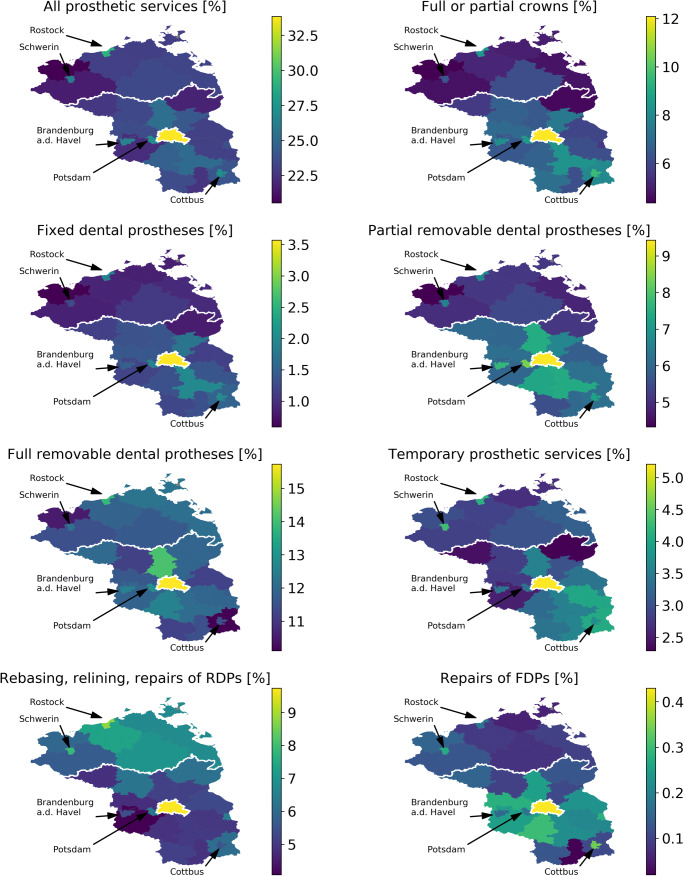
Regionally specific utilization of prosthetic services in Northeast Germany. Relative (in %) total (any) utilization and specific services utilization is shown. Larger cities with an increased or decreased utilization compared to the surrounding municipalities are further highlighted by arrows

Utilization was further different between different regions (Table 1, Fig. 2). Total (any) utilization of prosthetic services was significantly higher in Berlin than all other municipalities. Utilization was generally higher in cities than rural areas, and in the municipalities surrounding in Berlin compared with those in a larger distance to the capital. Utilization further differed geographically according to specific services. The utilization of crowns was especially high in Berlin, but also in large parts of Brandenburg and the major cities, but particularly low in most parts of Mecklenburg-Vorpommern. A similar pattern was apparent for FDPs and partial RDPs. In contrast, utilization between Brandenburg and Mecklenburg differed only limitedly for full RDPs. Berlin always showed a highest utilization.

Utilization of prosthetic services was assessed according to ICD-10 codes (Table 2). These were derived from outpatient diagnoses, as described. Utilization was higher for the majority of codes, e.g., for eye conditions (e.g., presbyopia or astigmatism), gonarthrosis, coxarthrosis, benign hypertension, hyperlipidemia, and hypercholesterinemia and unspecified chronic pain. The only code where utilization was significantly decreased was dementia. These patterns were apparent for different prosthetic services (Table 2).

**Table 2:** Utilization of prosthetic services according to International Disease Classification (ICD-10, German Modification) codes by the very old in Northeast Germany. Total (any) and specific services utilization (in %) is shown

ICD-10-GM	Outpatient diagnoses code	Description	N	%	Any utilization, N	%	Crowns, N	%	FDPs, N	%	Partial RDPs, N	%	Full RDPs, N	%	Temporary, N	%	RDP repair, N	%	FDP repair, N	%
–	Total	–	404.610	100.0	109.252	27.0	32.612	8.1	7.934	2.0	28.535	7.1	53.210	13.2	15.578	3.9	29.363	7.3	1.002	0.2
–	UUU	Special cases, without diagnostic certainty (e.g., passing on findings or replying to health insurance enquiries or order-related services)	374.109	92.5	107.057	28.6	32.273	8.6	7.854	2.1	28.168	7.5	52.211	14.0	15.260	4.1	28.565	7.6	993	0.3
Diseases of the circulatory system	I10.90	Essential hypertension, not further described	342.446	84.6	96.637	28.2	28.935	8.4	6.993	2.0	25.401	7.4	47.258	13.8	13.730	4.0	25.919	7.6	872	0.3
Factors influencing health status and contact with health services	Z25.1 *	The need for vaccination against influenza	258.991	64.0	77.670	30.0	23.354	9.0	5.593	2.2	20.564	7.9	38.131	14.7	10.694	4.1	20.588	7.9	721	0.3
Endocrine, nutritional and metabolic diseases	E11.90	Diabetes mellitus, type 2 without complications - Not designated as derailed	176.727	43.7	48.470	27.4	13.344	7.6	3.040	1.7	12.347	7.0	24.550	13.9	6.673	3.8	13.580	7.7	396	0.2
Diseases of the eye and adnex	H52.4	Presbyopia	169.474	41.9	58.555	34.6	19.739	11.6	4.944	2.9	16.503	9.7	28.140	16.6	8.376	4.9	14.700	8.7	620	0.4
Diseases of the eye and adnex	H52.2	Astigmatism	161.643	40.0	56.357	34.9	19.141	11.8	4.898	3.0	15.828	9.8	27.140	16.8	8.064	5.0	14.262	8.8	609	0.4
Diseases of the circulatory system	I25.9	Chronic ischaemic heart disease, not further specified	160.456	39.7	44.898	28.0	12.889	8.0	2.936	1.8	11.568	7.2	22.204	13.8	6.082	3.8	12.456	7.8	408	0.3
Diseases of the musculoskeletal system and connective tissue	M17.9	Gonarthrosis, not further described	141.200	34.9	45.678	32.3	14.503	10.3	3.622	2.6	12.537	8.9	22.332	15.8	6.352	4.5	12.444	8.8	480	0.3
Diseases of the circulatory system	I10.00	Benign essential hypertension - no indication of a hypertensive crisis	140.812	34.8	44.578	31.7	13.882	9.9	3.272	2.3	12.085	8.6	21.926	15.6	6.191	4.4	11.639	8.3	439	0.3
Diseases of the eye and adnex	H52.0	Accommodation disorders and refraction errors	139.335	34.4	48.946	35.1	16.742	12.0	4.219	3.0	13.800	9.9	23.615	16.9	6.997	5.0	12.194	8.8	517	0.4
Factors influencing health status and contact with health services	Z96.1 *	Presence of an intraocular lens implant	137.658	34.0	46.873	34.1	14.862	10.8	3.719	2.7	12.726	9.2	23.214	16.9	6.444	4.7	12.282	8.9	497	0.4
Diseases of the eye and adnex	H26.9	Cataract, not further specified	134.910	33.3	44.792	33.2	14.581	10.8	3.663	2.7	12.468	9.2	21.880	16.2	6.390	4.7	11.365	8.4	421	0.3
Symptoms, signs and abnormal clinical and laboratory findings, not elsewhere classified	R32 **	Unknown urinary incontinence	129.305	32.0	33.680	26.0	7.719	6.0	1.743	1.3	7.821	6.0	17.100	13.2	4.533	3.5	11.012	8.5	282	0.2
Mental and behavioral disorders	F03	Undescribed dementia	127.647	31.5	30.294	23.7	6.094	4.8	1.353	1.1	6.466	5.1	15.147	11.9	4.093	3.2	10.600	8.3	226	0.2
Symptoms, signs and abnormal clinical and laboratory findings, not elsewhere classified	R52.2 **	Other chronic pain	125.881	31.1	42.130	33.5	13.078	10.4	3.309	2.6	11.317	9.0	20.609	16.4	5.966	4.7	12.065	9.6	476	0.4
Diseases of the circulatory system	I50.9	Heart failure, not further specified	118.705	29.3	31.069	26.2	7.902	6.7	1.762	1.5	7.424	6.3	15.600	13.1	4.215	3.6	9.567	8.1	270	0.2
Endocrine, nutritional and metabolic diseases	E78.5	Hyperlipidemia, not further described	114.440	28.3	35.550	31.1	11.133	9.7	2.659	2.3	9.508	8.3	17.488	15.3	4.925	4.3	9.257	8.1	344	0.3
Diseases of the musculoskeletal system and connective tissue	M16.9	Coxarthrosis, not further described	112.428	27.8	36.003	32.0	11.311	10.1	2.729	2.4	9.848	8.8	17.718	15.8	4.922	4.4	9.816	8.7	366	0.3
Endocrine, nutritional and metabolic diseases	E78.0	Pure hypercholesterolemia	101.100	25.0	33.360	33.0	11.261	11.1	2.855	2.8	9.416	9.3	16.003	15.8	4.860	4.8	8.460	8.4	348	0.3
Diseases of the circulatory system	I70.9	Generalized and unspecified atherosclerosis	95.316	23.6	28.884	30.3	8.622	9.0	1.887	2.0	7.698	8.1	14.335	15.0	3.873	4.1	7.682	8.1	295	0.3
Diseases of the musculoskeletal system and connective tissue	M81.99	Osteoporosis, not further described - not further described Localization	95.150	23.5	29.927	31.5	9.031	9.5	2.118	2.2	8.007	8.4	14.678	15.4	3.974	4.2	8.422	8.9	339	0.4
Factors influencing health status and contact with health services	Z92.1 *	Long-term therapy (present) with anticoagulants in the patient’s own medical history	89.815	22.2	27.948	31.1	8.480	9.4	1.932	2.2	7.497	8.3	13.813	15.4	3.833	4.3	7.360	8.2	250	0.3
Diseases of the circulatory system	I83.9	Varices of the lower extremities without ulceration or inflammation	88.616	21.9	28.924	32.6	9.550	10.8	2.306	2.6	7.947	9.0	14.092	15.9	3.883	4.4	7.745	8.7	302	0.3
Endocrine, nutritional and metabolic diseases	E79.0	Hyperuricemia without signs of inflammatory arthritis or tophic gout	77.737	19.2	23.339	30.0	6.939	8.9	1.656	2.1	6.178	7.9	11.643	15.0	3.408	4.4	6.283	8.1	183	0.2
Diseases of the genitourinary system	N40	Prostatic hyperplasia	74.931	18.5	24.091	32.2	8.314	11.1	1.999	2.7	6.702	8.9	11.392	15.2	3.450	4.6	5.779	7.7	215	0.3

* Categories Z00-Z99 are intended for cases in which facts are indicated as “diagnoses” or “problems” which cannot be classified as disease, injury or external cause under categories A00-Y89

** This chapter includes (subjective and objective) symptoms, abnormal results of clinical or other investigations, and inaccurately identified conditions for which there is no classifiable diagnosis elsewhere

We further assessed the utilization of prosthetic services stratified according to different G-DRGs (Table 3). The following pattern emerged: Utilization was lower in participants hospitalized for severe chronic or respiratory conditions (chronic obstructive pulmonary condition, infections), cardiac insufficiency, renal insufficiency and urinary infections, and severe metabolic diseases. In contrast, utilization was higher for individuals hospitalized because of lens extraction, non-severe hypertension or cardiac arrhythmia or insufficiency, angioplasty or further invasive, non-severe cardiologic diagnosis, non-severe revision or replacement of the hip joint, or non-surgically treated diseases and injuries of the spinal column. Utilization was similar for participants hospitalized for esophagitis, gastroenteritis, gastrointestinal bleeding, ulcer disease, and various diseases of the digestive organs, as well as syncope, for example. Notably, the trends of higher or lower utilization were (again) largely uniform for all specific services except relining/rebasing/repair of RDPs, where trends were usually attenuated or even reversed (Table 3).

**Table 3:** Utilization of prosthetic services according to German Diagnosis related groups (G-DRG). Total (any) and specific services utilization (N; %) is shown

G-DRG	Description	N	%	Any utilization, N	%	Crowns, N	%	FDPs, N	%	Partial RDPs, N	%	Full RDPs, N	%	Temporary, N	%	RDP repair, N	%	FDP repair, N	%
–	Total population	404.610	100.0	109.252	27.0	32.612	8.1	7.934	2.0	28.535	7.1	53.210	13.2	15.578	3.9	29.363	7.3	1.002	0.2
F62B	Cardiac insufficiency and shock with extremely serious complications or comorbidity, with dialysis or complicated diagnosis or with certain high-level treatment or without complicated constellation, without specific high-level treatment, more than 1 day of occupancy in certain acute renal failure with extremely severe complications or comorbidity	40.295	10.0	9.124	22.6	1.885	4.7	378	0.9	1.942	4.8	4.624	11.5	1.116	2.8	3.064	7.6	65	0.2
G67C	Esophagitis, gastroenteritis, gastrointestinal hemorrhage, ulcer disease and various diseases of the digestive organs without certain or other complicating factors, without extremely severe complications or comorbidity	21.650	5.4	6.639	30.7	1.664	7.7	359	1.7	1.570	7.3	3.414	15.8	914	4.2	2.081	9.6	71	0.3
I41Z	Geriatric early rehabilitative complex treatment for diseases and disorders of the musculoskeletal system and connective tissue	21.131	5.2	6.550	31.0	1.745	8.3	430	2.0	1.667	7.9	3.347	15.8	1.008	4.8	1.907	9.0	75	0.4
K62B	Various metabolic diseases in paraplegia/tetraplegia or with complicated diagnosis or endoscopic insertion of a gastric balloon or age < 16 years, one occupancy day or without extremely severe complications or comorbidity or without certain costly/highly complex treatment	19.637	4.9	4.847	24.7	934	4.8	196	1.0	1.007	5.1	2.481	12.6	650	3.3	1.682	8.6	30	0.2
G67B	Esophagitis, gastroenteritis, gastrointestinal bleeding, ulcer disease and various diseases of the digestive organs with other complicating factors or with extremely severe complications or comorbidity	19.998	4.9	5.625	28.1	1.439	7.2	309	1.5	1.349	6.7	2.864	14.3	716	3.6	1.761	8.8	44	0.2
F71B	Non-severe cardiac arrhythmias and conduction disturbances without extremely severe complications or comorbidity or occupancy day, without catheter-assisted electrophysiological examination of the heart, without specific high-level treatment	16.666	4.1	5.541	33.2	1.774	10.6	421	2.5	1.486	8.9	2.692	16.2	783	4.7	1.429	8.6	51	0.3
F67D	Hypertension without complicated diagnosis, without extremely severe or severe complications or comorbidity, without certain moderately complex/complicated treatment, age > 17 years	15.300	3.8	4.854	31.7	1.319	8.6	311	2.0	1.250	8.2	2.454	16.0	667	4.4	1.308	8.5	44	0.3
E77I	Infections and inflammation of the respiratory system without complex diagnosis, without extremely severe complication or comorbidity or a complication or comorbidity, age > 0 years, except for para/quadriplegia, without complex treatment in multidrug-resistant pathogens	15.143	3.7	3.890	25.7	794	5.2	182	1.2	814	5.4	2.012	13.3	493	3.3	1.324	8.7	27	0.2
F48Z	Geriatric early rehabilitative complex treatment for diseases and disorders of the circulatory system	14.512	3.6	3.885	26.8	873	6.0	177	1.2	875	6.0	1.971	13.6	550	3.8	1.256	8.7	29	0.2
L63F	Infections of the urinary organs without extremely severe complications or comorbidity, without certain moderately costly / elaborate/highly costly treatment, without complex treatment multi-resistant pathogens (MRE), without certain serious infections, age > 5 and < 18 years, without severe complications or comorbidity or age > 17 and < 90 years	13.704	3.4	3.611	26.3	765	5.6	150	1.1	778	5.7	1.843	13.4	459	3.3	1.219	8.9	28	0.2
I68D	Non-surgically treated diseases and injuries of the spinal column, more than one occupancy day or other femoral fracture, without sacrum fracture, without certain moderately elaborate, elaborate or highly elaborate treatment	12.352	3.1	4.349	35.2	1.308	10.6	284	2.3	1.108	9.0	2.204	17.8	614	5.0	1.171	9.5	53	0.4
F73Z	Syncope and collapse	12.645	3.1	3.552	28.1	820	6.5	182	1.4	813	6.4	1.773	14.0	474	3.7	1.117	8.8	33	0.3
B80Z	Other head injuries	11.645	2.9	3.386	29.1	737	6.3	157	1.3	725	6.2	1.659	14.2	459	3.9	1.191	10.2	30	0.3
L60D	Renal insufficiency, more than one occupancy day, without dialysis, without extremely severe complications or comorbidity, age > 17 years or without severe complications or comorbidity, without complex intensive care treatment >196 /184/ - expense points	10.969	2.7	2.710	24.7	552	5.0	106	1.0	605	5.5	1.357	12.4	404	3.7	912	8.3	26	0.2
I47B	Revision or replacement of the hip joint without certain complicated factors, with complex diagnosis of the pelvis/thigh, with certain endoprosthetic or joint plastic surgery of the hip joint, with implantation or replacement of a radius head prosthesis.	10.975	2.7	3.645	33.2	1.174	10.7	293	2.7	1.000	9.1	1.809	16.5	519	4.7	890	8.1	35	0.3
J65Z	Injury of the skin, subcutis, and mamma	10.541	2.6	2.914	27.6	609	5.8	140	1.3	656	6.2	1.482	14.1	384	3.6	988	9.4	27	0.3
E65C	Chronic obstructive pulmonary disease without extremely severe complication or comorbidity, without complicated diagnosis, without FEV1 < 35% or a complication or comorbidity, age > 1 year, without specific moderately complex/expensive treatment	10.058	2.5	2.766	27.5	592	5.9	118	1.2	575	5.7	1.525	15.2	294	2.9	969	9.6	15	0.1
C08B	Extracapsular extraction of the lens (ECCE) without congenital malformation of the lens or certain interventions on the lens	9.574	2.4	3.264	34.1	954	10.0	238	2.5	890	9.3	1.623	17.0	454	4.7	946	9.9	28	0.3
A90A	Partial stationary geriatric complex treatment	9.831	2.4	3.510	35.7	1.031	10.5	246	2.5	953	9.7	1.766	18.0	515	5.2	943	9.6	46	0.5
E69B	Bronchitis and bronchial asthma, more than 1 day of treatment Age > 55 years or with extremely severe or severe complication or comorbidity, age > 0 years or 1 day of treatment or without extremely severe or severe complication or comorbidity, age < 1 year or flexible bronchoscopy, age < 16 years or determined moderate treatment, with RS virus -Infection.	9.830	2.4	2.590	26.3	543	5.5	125	1.3	540	5.5	1.305	13.3	338	3.4	878	8.9	16	0.2
F49G	Invasive cardiological diagnosis except in acute myocardial infarction, without extremely severe complication or comorbidity, age > 17 years, without cardiac mapping, without severe complication or comorbidity at day of treatment>1, without complex diagnosis, without specific intervention	9.777	2.4	4.005	41.0	1.522	15.6	372	3.8	1.239	12.7	1.901	19.4	552	5.6	952	9.7	55	0.6
I34Z	Geriatric early rehabilitative complex treatment with specific operating room procedure for diseases and disorders of the musculoskeletal system and connective tissue	9.419	2.3	2.752	29.2	717	7.6	162	1.7	688	7.3	1.405	14.9	419	4.4	794	8.4	34	0.4
F62D	Cardiac insufficiency and shock without extremely serious complications or comorbidity or without dialysis, without complicated diagnosis, without complicated constellation, without specific high-level treatment, 1 day of occupancy	8.615	2.1	2.611	30.3	621	7.2	139	1.6	621	7.2	1.355	15.7	323	3.7	792	9.2	21	0.2
B70B	Apoplexy with neurological complex treatment of acute stroke, more than 72 h, without complicated diagnosis or with complex cerebrovascular vasospasm or intensive care complex treatment	8.499	2.1	2.255	26.5	525	6.2	108	1.3	502	5.9	1.149	13.5	297	3.5	690	8.1	13	0.2
L64A	Other urinary organ diseases with extremely severe or severe complications or comorbidity or certain diagnosis, more than one occupancy day or urethra-cystoscopy, congenital malformation or age < 3 years	5.669	1.4	1.538	27.1	379	6.7	76	1.3	348	6.1	792	14.0	212	3.7	437	7.7	11	0.2

In multivariable analysis (Table S1), gender was minimally, but significantly associated with utilization (OR; 95% CI: 0.95; 0.93–0.98). Social hardship status (1.19; 1.17–1.21), federal state (Brandenburg 0.57; 0.56–0.59; Mecklenburg-Vorpommern: 0.66; 0.64–0.67) and age significantly affected utilization (0.95; 0.95–0.95 per year of age). Moreover, some specific diagnoses, namely head injury (1.3; 1.1–1.5) or non-severe arrhythmia (1.1; 1.0–1.2) were significantly associated with utilization, while the magnitude of association was generally limited (Table S1). Pseudo-R^2^ indicated that the model generally had extremely limited explanatory power (at R^2^ = 0.03).

## Discussion

A range of aspects affect the quantity and quality (number and type) of healthcare utilization, with patient characteristics (including income and education, financial means, place of living, and attitude) and needs (determined by age, gender, and illnesses) on the one side and provider and system features (including physical, financial, and organizational access and care quality) on the other side [17, 18]. These factors have also been identified to underlie oral healthcare utilization [19]. As low utilization of healthcare is associated with late presentation to care, and lack of treatment, both resulting in poor health outcomes and health disparities, exploring such aspects is relevant [20].

In the present study, we hypothesized that the utilization of prosthetic services in the very old was affected by age, morbidity, socioeconomic status, place of living, and that disparities between groups would be services specific. Using claims data from one of the largest insurers in Germany, we assessed these utilization patterns. Our analysis showed that no significant differences in prosthetic services usage occurred between genders (at least not in univariate analyses), while the utilization significantly decreased with age, and was also generally lower in rural areas than conurbations. The prosthetic services most frequently utilized up to a certain age were crowns and full RDPs; in very high age, repairs, relining or rebasing (mainly of RDPs) dominated. Our evaluation of prosthetic services utilization according to DRGs demonstrated increased utilization for DRGs coding for largely “less severe” and more common diseases among elderly (like conditions of the eye or the hip joint, as well as non-severe hypertension or cardiac arrhythmia or insufficiency), and drastically reduced utilization for those with severe, often life-threatening disease (like renal or cardiac failure). A similar pattern emerged from subgrouping utilization according to ICD-10 codes, with increased utilization for many of these, and nearly halved utilization in those elderly with dementia. Notably, these ICD-10 codes were derived from ambulatory (mainly general practitioner, GP) diagnoses, with most individuals obviously being capable of attending the GP (in contrast to DRGs, which are associated with in-patient hospitalization). These findings are only applicable to individuals from Northeast Germany, as laid out further down below.

Our analysis reflects the reported poor oral health of the very old in Northeast Germany. While there is trend of fewer missing teeth, fewer full RDPs and more FDPs in seniors in many high-income countries [21], the very old, especially if residing in long-term care, experience a significant lack of dental therapy. A large share, in some cases even the majority of this group have been found to require tooth extractions, and repair, rebasing or relining RDPs [19, 22]. Age is associated with an increasing frequency of chronic diseases, disablement or hospitalization [23]. German social and healthcare law has been prioritizing those with chronic diseases [24]. This regulation, however, does not consider poor oral health at all, and does not account for any interplay between dental and general health. Policy makers may want to revisit this regulation to better integrate dental health and the associated treatment need and service provision. This may be done, for example, by cross-discipline screening programs (e.g., GP screening for oral conditions, and dentists for general ones, accounting for the fact that utilization patterns are likely to differ between different diagnoses, with some individuals visiting the dentist more often than the GP and vice versa). Interprofessional care may allow to account for the described interlinkage between oral and systemic health better (e.g., poor masticatory performance impacting on nutritional status, poorly managed periodontal disease impacting on diabetes mellitus and vice versa). Chronically ill individuals should further be prioritized to receive dental and, specifically, prosthetic therapy without additional costs, as these have been found to reduce the uptake of (required) care (see below).

A number of studies found that women use (mainly outpatient) healthcare more frequently than men, largely due to a difference in general health status [25, 26]. It has also been found that women have better oral health and lower treatment needs than men [19]. This association was only confirmed by our study in multivariate analyses, while overall, the impact of gender on prosthetic service utilization was limited.

Financial aspects have been found a main barrier for healthcare utilization. These are inherent in the organization and structure of the healthcare system; they are both measurable and modifiable and can thus be addressed to increase utilization [20]. In the very old, specifically, insurance coverage has been found a most relevant factor for healthcare and, specifically, dental services utilization in many countries, mainly with adults being insured via their employer as long as they work, and then loosing this coverage at retirement [27–29]. Both having dental care coverage and having access to a dentist have been found to drastically increase dental care utilization [30], and factors associated with financial capabilities (like education or income) also determine prosthetic service pattern (e.g., replacement of teeth using FDPs vs. RDPs) [31]. We confirm these findings, with a significant increase in utilization for those with social hardship status, where costs are usually fully covered by the insurance and out-of-pocket expenses avoided. As this group, usually with low socioeconomic and educational status, also shows poorest oral health [32], this political measure seems to successfully tackle health disparities, and may be adopted in other healthcare systems. Further studies should explore the specific services most affected by this regulation and may aim to link utilization data with oral health data, allowing to compare epidemiologically demonstrated needs with provided therapies.

Another structural barrier to care utilization is place of living [20]. There are great disparities in medical care utilization between rural areas, which are increasingly underserviced in many high-income countries (Germany among them), and urban areas, where provider clustering facilitates supply-side-induced demand. In our analysis in Northeast Germany, the more rural federal states Brandenburg and Mecklenburg-Vorpommern showed much lower dentist densities (around 80–90 dentists per 100,000 inhabitants in 2016) than Berlin, which has the highest density of all federal states in Germany (at around 120:100,000) [33]. Both the physical access (time and costs of travel, availability of public transport) to dental practices and the provision of dental care to the very old residing in long-term care facilities will be associated with dentist availability [34, 35]. More detailed spatial analyses may allow to understand distinct factors behind these geographic disparities, and further political efforts may be needed to provide sufficient prosthetic services in more rural areas.

We found that the described disparities in utilization are specific for different prosthetic services. For example, with age, the utilization of crowns and FDPs decreased dramatically, while this was less clear for RDPs and not at all detectable for repairs. It seems that services targeting the maintenance of prostheses are prioritized in higher age. Similar findings emerged when services were stratified according to ICD-10 or DRG codes, as discussed above. It would be relevant to contrast these findings against the utilization of other dental services (e.g., restorative or periodontal care), and to explore if the provided services are, again, needs-adequate.

This study has a number of strengths and limitations. First, this is so far the only available analysis focusing on prosthetic services pattern in very old Germans. Our study builds on a large, diverse sample from different federal states. Stratified analyses according to DRGs and ICD-10 have not yet been performed when evaluating dental healthcare and may yield useful insights for policy makers and future research. Second, and as a limitation, the used data suffer from the described weaknesses of claims data, and do not allow to infer on oral health status of the very old. They also do not permit analyses to explore causality, and given the way data on prosthetic services utilization were available, we are unable to assess the specific extend of the services provided (e.g., how many teeth were replaced by an FDP or RDP). For such analysis, more granular fee-item data would be required. Third, our analyzes were largely descriptive, and the provided bivariate analysis are prone for confounding. This is why we performed multivariable analysis, which, however, largely confirmed the findings from less elaborate evaluations. Notably, the logistic regression model showed only low predictive power when applying it to a hold-out test set despite high classification accuracy (above 90%), and came with low explanatory value. This may be, as a range of relevant confounders (e.g., medication, care status) were not available and accounted for. Last, the analyzed sample was not representative of the entire German population. The Northeast of Germany is over-proportionally old (around 23% of those insured within AOK Nordost fell into our group of “very old” individuals; this proportion is nearly halved on a national level), economically less prosperous (as described), has suffered from larger shares of the population moving to other areas of Germany within the last 30 years, and shows a lower proportion of migrants who immigrated to Germany in the 1960s, largely as most of the Northeast belonged to the former German Democratic Republic at that time. All of this will affect service utilization, but also morbidity. For example, the relevance of any kind of hardship status may be different elsewhere in Germany. Moreover, the differences in the levels of urbanization between Berlin and the other two states are larger than in any other region of Germany, which is why the found differences in our study may be rather extreme than the standard in Germany. As described, those insured by the AOK do not represent the socio-economic cross-section of Germany, with more affluent people being more often privately insured or may choose other insurers with more attractive additional insurance options. Again, service utilization and patterns will differ here (e.g., the number of retained teeth and hence number of provided FDPs versus RDPs will be driven by such socioeconomic factors) [11, 32].

In conclusion, and within the limitations of this claims-data-based study, the utilization of prosthetic services in the very old in Northeast Germany showed significant disparities within populations and service types. With increasing age, the overall utilization decreased for nearly all services except prostheses maintenance. General health was significantly associated with prosthetic services utilization, with more common and less severe diagnoses or treatments oftentimes being associated with increased or unaffected care usage, while life-threatening or mental diseases negatively affect utilization. Utilization was significantly higher in urban versus rural areas. Reducing financial barriers by providing hardship assistance for those with low income significantly increased utilization. There seems to be great need to better understand the drivers of utilization, and to develop and evaluate interventions to maintain sufficient prosthetic care up to high age.

## Electronic supplementary material

ESM 1 (DOCX 25 kb)
