# Supplementary material for: Prosthetic treatment patterns in the very old: an insurance database analysis from Northeast Germany
Source: Clin Oral Investig. 2020 Apr 17;24(11):3981–95. doi: 10.1007/s00784-020-03264-x (PMC7544711; doi:10.1007/s00784-020-03264-x)
Supplement: Supplementary file 1 — (DOCX 25 kb) [file 784_2020_3264_MOESM1_ESM.docx]

**Appendix**

Table S1: Factors associated with the total (any) utilization of prosthetic services in the very old in Northeast Germany 2012.

| **Covariate** |  | **2.5%** | **97.5%** | **OR** |
| --- | --- | --- | --- | --- |
| Intercept |  | 5.454 | 8.05 | 6.626 |
| Gender [male] |  | 0.928 | 0.98 | 0.954 |
| Deceased [yes] |  | 0.514 | 0.577 | 0.545 |
| Social hardship status [yes] |  | 1.165 | 1.218 | 1.191 |
| Federal state [Brandenburg] |  | 0.557 | 0.587 | 0.572 |
| Federal State [Mecklenburg-Vorpommern] |  | 0.638 | 0.674 | 0.656 |
| Age |  | 0.949 | 0.953 | 0.951 |
| A90A | Partial stationary geriatric complex treatment | 0.964 | 1.262 | 1.103 |
| B70B | Apoplexy with neurological complex treatment of acute stroke, more than 72 hours, without complicated diagnosis or with complex cerebrovascular vasospasm or intensive care complex treatment | 0.786 | 1.118 | 0.937 |
| B80Z | Other head injuries | 1.111 | 1.5 | 1.291 |
| C08B | Extracapsular extraction of the lens (ECCE) without congenital malformation of the lens or certain interventions on the lens | 0.879 | 1.085 | 0.977 |
| E65C | Chronic obstructive pulmonary disease without extremely severe complication or comorbidity, without complicated diagnosis, without FEV1 <35% or a complication or comorbidity, age> 1 year, without specific moderately complex / expensive treatment | 0.873 | 1.093 | 0.977 |
| E69B | Bronchitis and bronchial asthma, more than 1 day of treatment Age> 55 years or with extremely severe or severe complication or comorbidity, age> 0 years or 1 day of treatment or without extremely severe or severe complication or comorbidity, age <1 year or flexible bronchoscopy, age <16 years or determined moderate treatment, with RS virus -Infection. | 0.869 | 1.202 | 1.022 |
| F48Z | Geriatric early rehabilitative complex treatment for diseases and disorders of the circulatory system | 0.816 | 1.089 | 0.943 |
| F49G | Invasive cardiological diagnosis except in acute myocardial infarction, without extremely severe complication or comorbidity, age> 17 years, without cardiac mapping, without severe complication or comorbidity at day of treatment> 1, without complex diagnosis, without specific intervention | 0.9 | 1.254 | 1.062 |
| F62B | Cardiac insufficiency and shock with extremely serious complications or comorbidity, with dialysis or complicated diagnosis or with certain high-level treatment or without complicated constellation, without specific high-level treatment, more than 1 day of occupancy in certain acute renal failure with extremely severe complications or comorbidity | 0.909 | 1.021 | 0.963 |
| F67D | Hypertension without complicated diagnosis, without extremely severe or severe complications or comorbidity, without certain moderately complex / complicated treatment, age> 17 years | 0.894 | 1.102 | 0.993 |
| F71B | Non-severe cardiac arrhythmias and conduction disturbances without extremely severe complications or comorbidity or occupancy day, without catheter-assisted electrophysiological examination of the heart, without specific high-level treatment | 0.999 | 1.189 | 1.09 |
| F73Z | Syncope and collapse | 0.801 | 1.02 | 0.904 |
| G67B | Esophagitis, gastroenteritis, gastrointestinal bleeding, ulcer disease and various diseases of the digestive organs with other complicating factors or with extremely severe complications or comorbidity | 0.878 | 1.066 | 0.967 |
| G67C | Esophagitis, gastroenteritis, gastrointestinal hemorrhage, ulcer disease and various diseases of the digestive organs without certain or other complicating factors, without extremely severe complications or comorbidity | 0.986 | 1.186 | 1.081 |
| I34Z | Geriatric early rehabilitative complex treatment with specific operating room procedure for diseases and disorders of the musculoskeletal system and connective tissue | 0.762 | 1.111 | 0.92 |
| I41Z | Geriatric early rehabilitative complex treatment for diseases and disorders of the musculoskeletal system and connective tissue | 0.882 | 1.088 | 0.98 |
| I47B | Revision or replacement of the hip joint without certain complicated factors, with complex diagnosis of the pelvis/thigh, with certain endoprosthetic or joint plastic surgery of the hip joint, with implantation or replacement of a radius head prosthesis. | 0.842 | 1.103 | 0.963 |
| J65Z | Injury of the skin, subcutis and mamma | 0.867 | 1.199 | 1.02 |
| K62B | Various metabolic diseases in paraplegia / tetraplegia or with complicated diagnosis or endoscopic insertion of a gastric balloon or age < 16 years, one occupancy day or without extremely severe complications or comorbidity or without certain costly / highly complex treatment | 0.896 | 1.122 | 1.002 |
| L60D | Renal insufficiency, more than one occupancy day, without dialysis, without extremely severe complications or comorbidity, age > 17 years or without severe complications or comorbidity, without complex intensive care treatment > 196 / 184 / - expense points | 0.752 | 1.025 | 0.878 |
| L63F | Infections of the urinary organs without extremely severe complications or comorbidity, without certain moderately costly / elaborate / highly costly treatment, without complex treatment multi-resistant pathogens (MRE), without certain serious infections, age > 5 and < 18 years, without severe complications or comorbidity or age > 17 and < 90 years | 0.824 | 1.064 | 0.936 |
| L64A | Other urinary organ diseases with extremely severe or severe complications or comorbidity or certain diagnosis, more than one occupancy day or urethra-cystoscopy, congenital malformation or age < 3 years | 0.832 | 1.134 | 0.971 |
| E11.90 | Diabetes mellitus, type 2 without complications - Not designated as derailed | 0.981 | 0.989 | 0.985 |
| E78.0 | Pure hypercholesterolemia | 1.005 | 1.019 | 1.012 |
| E78.5 | Hyperlipidemia, not further described | 0.994 | 1.008 | 1.001 |
| E79.0 | Hyperuricemia without signs of inflammatory arthritis or tophic gout | 0.983 | 0.999 | 0.991 |
| F03 | Undescribed dementia | 0.994 | 1.01 | 1.002 |
| H26.9 | Cataract, not further specified | 0.99 | 1.004 | 0.997 |
| H52.0 | Accommodation disorders and refraction errors | 0.998 | 1.023 | 1.01 |
| H52.2 | Astigmatism | 1.005 | 1.031 | 1.017 |
| H52.4 | Presbyopia | 1.01 | 1.035 | 1.022 |
| I10.00 | Benign essential hypertension - no indication of a hypertensive crisis | 0.997 | 1.012 | 1.004 |
| I10.90 | Essential hypertension, not further described | 1.005 | 1.013 | 1.009 |
| I25.9 | Chronic ischaemic heart disease, not further specified | 0.99 | 1.001 | 0.996 |
| I50.9 | Heart failure, not further specified | 0.983 | 1 | 0.992 |
| I70.9 | Generalized and unspecified atherosclerosis | 0.983 | 1.004 | 0.994 |
| I83.9 | Varices of the lower extremities without ulceration or inflammation | 1 | 1.015 | 1.008 |
| M16.9 | Coxarthrosis, not further described | 0.996 | 1.01 | 1.003 |
| M17.9 | Gonarthrosis, not further described | 1.012 | 1.023 | 1.017 |
| M81.99 | Osteoporosis, not further described - not further described Localization | 1.02 | 1.034 | 1.027 |
| N40 | Prostatic hyperplasia | 1.023 | 1.038 | 1.031 |
| R32 ** | Unknown urinary incontinence | 1.008 | 1.027 | 1.017 |
| R52.2 ** | Other chronic pain | 1.02 | 1.04 | 1.03 |
| UUU | Special cases, without diagnostic certainty (e.g. passing on findings or replying to health insurance enquiries or order-related services) | 1.017 | 1.025 | 1.021 |
| Z25.1 * | The need for vaccination against influenza | 1.056 | 1.097 | 1.076 |
| Z92.1 * | Long-term therapy (present) with anticoagulants in the patient's own medical history | 0.99 | 1.004 | 0.997 |
| Z96.1 * | Presence of an intraocular lens implant | 1.027 | 1.044 | 1.035 |

| * Categories Z00-Z99 are intended for cases in which facts are indicated as "diagnoses" or "problems" which  cannot be classified as disease, injury or external cause under categories A00-Y89. |
| --- |
| ** This chapter includes (subjective and objective) symptoms, abnormal results of clinical or other investigations,  and inaccurately identified conditions for which there is no classifiable diagnosis elsewhere. |
